# Polarized monocyte response to cytokine stimulation

**DOI:** 10.1186/gb-2005-6-2-r15

**Published:** 2005-01-21

**Authors:** Dirk Nagorsen, Sara Deola, Kina Smith, Ena Wang, Vladia Monsurro, Paola Zanovello, Francesco M Marincola, Monica C Panelli

**Affiliations:** 1 Immunogenetics Section, Department of Transfusion Medicine, Clinical Center, National Institutes of Health, Bethesda, MD 20892-1502, USA; 2 Department of Oncology and Surgical Sciences, Oncology Section, University of Padova, Padova 35100, Italy; 3 Current address: Charité - Universitätsmedizin Berlin, Campus Benjamin Franklin, Medizinische Klinik III, Hindenburgdamm 30, 12200 Berlin, Germany

## Abstract

**Background:**

Mononuclear phagocytes (MPs) stand at the crossroads between the induction of acute inflammation to recruit and activate immune effector cells and the downmodulation of the inflammatory process to contain collateral damage. This decision is extensively modulated by the cytokine microenvironment, which includes a broad array of cytokines whose direct effect on MPs remains largely unexplored. Therefore, we tested whether polarized responses of MPs to pathogens are related to the influence of selected cytokines or represent a mandatory molecular switch through which most cytokines operate.

**Results:**

Circulating CD14^+ ^MPs were exposed to bacterial lipopolysaccharide (LPS) followed by exposure to an array of cytokines, chemokines and soluble factors involved in the immune response. Gene expression was studied by global transcript analysis. Two main classes of cytokines were identified that induced a classical or an alternative pathway of MP activation. Expression of genes affected by NFκB activation was most predictive of the two main classes, suggesting that this pathway is a fundamental target of cytokine regulation. As LPS itself induces a classical type of activation, the most dramatic modulation was observed toward the alternative pathway, suggesting that a broad array of cytokines may counteract the pro-inflammatory effects of bacterial components.

**Conclusions:**

This analysis is directly informative of the primary effect of individual cytokines on the early stages of LPS stimulation and, therefore, may be most informative of the way MP maturation may be polarized at the early stages of the immune response.

## Abstract 2

A comprehensive study of the transcriptional response of mononuclear phagocytes to cytokines reveals distinct classes of cytokines that elicit either the classical or alternative pathway of monocyte activation.

## Background

Resident and recruited mononuclear phagocytes (MPs) display a versatile phenotype that reflects the plasticity of these cells in response to microenvironmental signals. This heterogeneity spans a continuous spectrum that can be polarized into two extremes recently described by Mantovani *et al*. [1]. Pathogen stimulation exemplified, for instance, by lipopolysaccharide (LPS) stimulation in the presence of interferon (IFN)-γ induces M1 MPs through engagement of Toll-like receptors (TLRs). M1 MPs are true antigen-presenting cells capable not only of killing invading organisms but of concomitantly recruiting and activating immune effector cells [2,3]. Treatment of MPs with type II cytokines such as interleukin (IL)-4, IL-13 and, partly, IL-10 [4], polarizes their function towards tissue repair, angiogenesis and containment of collateral damage through reduction of inflammation (the M2 macrophage phenotype). This alternative mode of macrophage activation accounts for a distinct phenotype with a key role in humoral immunity and tissue repair [5].

It has been suggested that the extreme dichotomy between a classical M1 and an alternative M2 polarization of macrophage function may not take into account intermediate regulation by cytokines such as IL-10, transforming growth factors (TGF-α and TGF-β), macrophage colony-stimulating factor (M-CSF), IFN-α and IFN-β and tumor necrosis factor (TNF) [5]. Most important, a rigid dichotomy in MP function may not directly apply to physiological and/or pathological conditions in which these cells are exposed to an array of cytokines produced by innate or adaptive immune mechanisms during infection, tissue damage or in other conditions. Indeed, a comprehensive overview of the modulatory properties of cytokines on the MP reaction to a pathogen is missing. In addition, little is known about the transcriptional changes occurring in MPs on exposure to pathogen components such as LPS.

A recent study analyzed the transcriptional profile induced by the exposure of circulating MP conditioned *in vitro *for 7 days with IL-4 and GM-CSF (immature dendritic cell, (DC)) to pathogen components [6]. Bacterial, viral and fungal components elicited distinctive pathways that were, however, largely overlapping. The predominant response of these DCs to most pathogen components encompassed a rapid upregulation of genes associated with the innate arm of the immune response followed by induction of adaptive immune response genes.

The response of circulating MP-derived DCs is short-lived as these cells can exhaust their production of effector molecules (cytokines and chemokines) within a few hours of LPS stimulation [7]. The transience of mRNA and protein expression can cause DCs to redirect the immune response in different ways at different time points. For instance, soon after stimulation, DCs elicit T-cell responses of the Th-1 type, whereas at later stage of activation they prime T-cell responses of the Th-2 type, suggesting that their function is strongly dependent on the timing and duration of exposure to individual and/or combined stimulatory conditions in the surrounding microenvironment. At the transcriptional level, the dual function of DCs shifted from an early pre-inflammatory phase occurring within 3 hours to a later regulatory phase occurring approximately 8 hours following LPS exposure. During these evolving stages of DC activation, cytokines play a dominant role in shaping the function of DCs and other immune cells, providing a malleable link between the innate and adaptive immune responses [8]. In natural conditions, circulating or resident MPs may encounter a pathogen before the surrounding microenvironment has a chance to influence their maturation. Therefore, it is unknown whether non-conditioned circulating CD14^+ ^MPs would react similarly to DCs on engagement with infectious agents. Thus, a preliminary aim of this study was to evaluate the kinetics of the response of non-conditioned circulating CD14^+ ^MP to LPS. The results suggested that these cells respond to LPS similarly to immature CD14^- ^DCs, with a surge in transcriptional activity that peaks around 3 hours after stimulation and in which the activation of genes associated with a classical activation of innate immune mechanisms predominates [6].

The stringent dichotomy describing a classical activation of MPs into mature antigen-presenting cells caused by IFN-γ and an alternative induction into macrophages induced by IL-4 and IL-13 may not apply to physiological conditions in which the microenvironment responds to pathogen exposure with a broad array of cytokine secretion. We therefore investigated whether polarized responses of MPs to pathogens are extreme behaviors that can be observed *in vitro *by studying a few illustrative cytokines or whether they represent a mandatory molecular switch through which most cytokines operate. Thus, we stimulated non-conditioned CD14^+ ^MPs with LPS for 1 hour. The MPs were then exposed to an array of different cytokines that may be expressed in distinct pathologic conditions by different immune-cell subsets. The 1-hour interval was empirically selected to induce a biphasic model in which the presumed modulation by cytokines occurred during an ongoing reaction to LPS. This allowed mapping of cytokines into conditional subclasses based on their effects on the global transcriptional changes responsible for MP activation and differentiation.

Two main classes of cytokines were identified that induced a classical or alternative pathway of MP activation, respectively. An intermediate class (including IL-10) was also identified, while TNF-α, TNF-β and GM-CSF displayed a quite distinct behavior from the other cytokines. Expression of genes affected by NFκB activation was most predictive of the two main classes, suggesting that in most cases the NFκB pathway is a central target of cytokine regulation that modulates the cascade of events following LPS stimulation. Overall, it seems that MP maturation/differentiation goes through a molecular switch that is partly independent of the fine differences in the stimulatory properties of the various cytokines and, with few exceptions, is pre-programmed towards a classical or an alternative route. As LPS itself induces a classical type of activation, the most dramatic modulation in this model was observed toward the alternative pathway, suggesting that a broad array of cytokines may counteract the pro-inflammatory effects of bacterial components.

## Results

### Effect of LPS stimulation on circulating CD14^+ ^MPs

Enriched MP preparations (about 90% CD14^+^) were exposed to LPS. Total RNA extracts were obtained 4 and 9 hours after. These time points were selected to catch salient stages of the biphasic response of MP to LPS described by others [6,7]. Amplified antisense RNA (aRNA) [9] was hybridized to a custom-made 17,000 (17K)-clone cDNA microarray chip enriched with genes relevant to immune function. The transcriptional profile of LPS-induced MPs was similar to that described by others in DCs [6]. In particular, genes associated with the innate response of CD14^-^, immature DCs to pathogen components [6,10] were similarly upregulated in CD14^+ ^MPs (data not shown). This finding suggests that the differential expression of the LPS co-receptor CD14 between the two cell populations has a relatively minor impact on the transcriptional regulation of the innate immune response [11].

### Kinetics of the response of CD14^+ ^MPs to LPS and its modulation by cytokines

**Figure 1:**
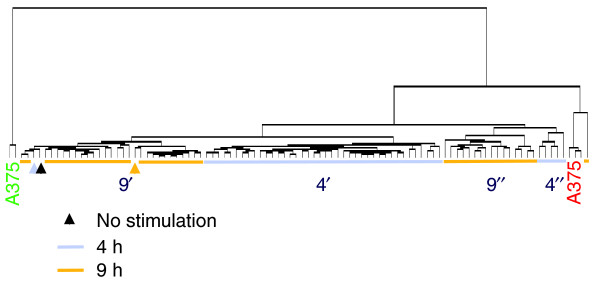
Unsupervised clustering of LPS-stimulated CD14^+ ^MPs exposed to distinct cytokine treatments. CD14^+ ^MPs were stimulated in parallel with LPS and exposed after 1 h to 42 individual cytokines (see Table 1 for cytokines used). Antisense RNA obtained 4 and 9 h following LPS stimulation was hybridized to custom-made 17K cDNA arrays. Unsupervised Eisen clustering [12] was applied to the complete, unfiltered dataset of 98 experiments. Arrowheads represent control samples that did not receive any stimulation and were obtained at different time points to parallel culture conditions during stimulation (0, 4 and 9 h). Light blue and gold represent samples obtained 4 and 9 h after stimulation, respectively and black represents no stimulation. A375 is a melanoma cell line that was used for quality control alternating conventional (Cy5, red) or reciprocal (Cy3, green) labeling every 25 experiments as previously described [9]. Experiments cluster closer together according to time rather than type of stimulation, with samples obtained after 4 h clustering together (4') with the exception of few cytokines (4"). With few exceptions (9"), cytokine treatments at 9 h clustered together with non-stimulated MPs (9').

Aliquots of CD14^+ ^MPs were stimulated in parallel with LPS and exposed 1 hour later to individual cytokines selected from a library of recombinant proteins possibly relevant to MP regulation. MPs were kept in culture for 4 and 9 hours, at which times aRNA was prepared for transcriptional analysis. Unsupervised Eisen's clustering [12] was applied to the complete dataset (Figure 1). The kinetics of the response to LPS had the greatest influence on the global transcriptional profile of MP induction; samples preferentially clustered according to time of stimulation rather than type of treatment. This was underlined by the observation that MPs stimulated with LPS alone clustered with the cytokine-stimulated MPs according to the time elapsed after stimulation. In addition, a cluster containing most of the samples obtained 9 hours after stimulation (9') included three control samples consisting of CD14^+ ^MPs not exposed to LPS or cytokines (no stimulation). These three samples were prepared at times 0, 4 and 9 hours to parallel the culture conditions used for stimulation. This finding suggested that the transcriptional profile of CD14^+ ^MPs 9 hours after LPS stimulation and 8 hours after treatment with most cytokines converges toward a less reactive metabolic state closer to that of unstimulated MPs. Several cytokines, however, maintained a more active metabolic profile and after 9 hours retained a transcriptional footprint relatively close to that of samples treated for 4 hours (9"). This group included the genes for most IFN-α isoforms, IFN-β, vascular endothelial growth factor (VEGF), FLT-3 ligand, TGF-α, the chemokine RANTES (CCL5), IL-2, IL-4, IL-15 and the chemokines MIP1α (CCL3) and MIP1β (CCL4), suggesting that these cytokines may have relatively prolonged kinetics of MP activation.

The average number of genes whose expression was increased compared to unstimulated MPs was higher (420 genes) after 4 hours than after 9 hours (265 genes). About half of the genes upregulated after 4 hours remained upregulated at 9 hours (223 genes). This is in accordance with Huang's observation [6] that transcriptional changes in DCs occur predominantly in the early phase of the response to pathogen components, with about half the genes displaying transitory expression and the other half sustained expression. For this reason, subsequent analyses were limited to the 4-hour time point.

### Transcriptional modulation by cytokines 4 hours after LPS stimulation of CD14^+ ^MPs

**Figure 2:**
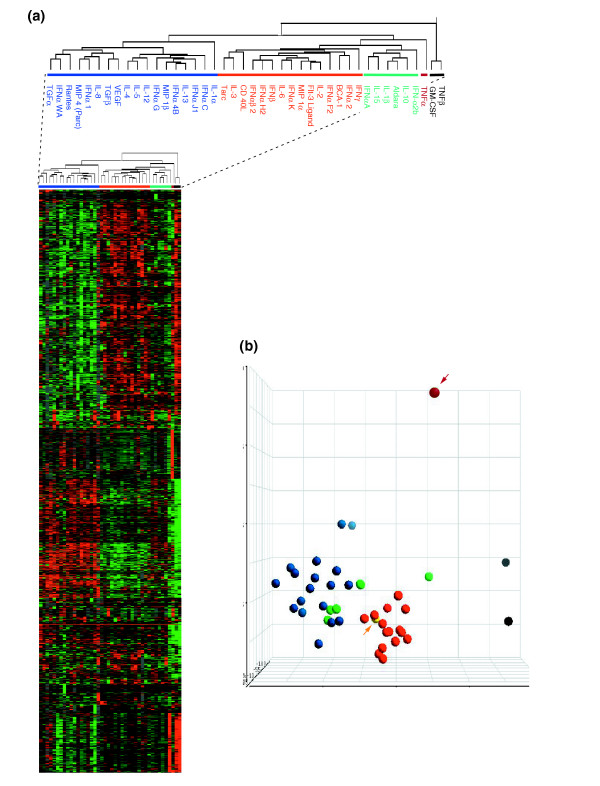
Definition of cytokine classes based on their modulatory effect on the response of CD14+ circulating MP to LPS. **(a) **Definition of cytokine classes based on their modulatory effect on the response of CD14^+ ^circulating MPs to LPS. CD14^+ ^MPs were stimulated with LPS and exposed after 1 h to 42 individual cytokine stimulations. The clusterogram represents 2,057 genes obtained by Eisen hierarchical clustering of the complete 17K dataset filtered for genes that are expressed in a minimum of 80% of the samples 4 h after LPS stimulation, and that, at least in one experiment, displayed a greater than threefold change in expression over stimulation with LPS alone. Two main classes of cytokines are distinguished: the blue bar indicates type II cytokines (for example, IL-4 and IL-13) and the red bar indicates type I cytokines (such as IFN-γ, CD40L and FLT-3L). A smaller third class including IL-10 is also shown (green bar). TNF-α (purple bar), TNF-β and GM-CSF (dark-gray bars) clustered separately from all other cytokines. **(b) **Unsupervised principal component analysis (PCA) of the unfiltered 17K gene dataset. Cytokine treatments are color-coded according to the groups classified in (a): blue circles, alternative type I cytokines; green circles, alternative type II cytokines; red circles, classical cytokines; purple circle (arrowed), TNF-α; dark-gray circles, TNF-β and GM-CSF; yellow circle (arrowed), LPS alone.

Although LPS alone strongly affected the transcriptional program of MPs, it was possible to discern the contribution of individual cytokines. This analysis was limited to samples treated for 4 hours, when the most dramatic effects on gene modulation were noted. Genes differentially expressed in samples treated with cytokines compared to those treated with LPS alone were identified. Stringent criteria were applied to select genes expressed in at least 80% of samples with a threefold or greater increase or reduction in expression over LPS in at least one of the cytokine-treated samples. This gave 2,057 genes that were deemed most relevant to the analysis. Using these genes, all cytokine-treated samples were subjected to unsupervised clustering to evaluate their relatedness (Figure 2a).

Two main classes of cytokines were identified. One class (Figure 2a, blue horizontal line) included IL-4, IL-13 TGF-α, TGF-β and VEGF, which are unquestionably associated with the alternative pathway of MP activation [1,5]. The second class (Figure 2a, red line) included IFN-α2, IFN-β, IFN-γ, CD40 ligand (CD40L), and FLT-3 ligand, which are generally associated with the classical pathway of MP activation [2,3]. Therefore, we considered the first cluster representative of the alternative and the second of the classical pathway of MP activation in response to LPS. As predicted by Gordon [5], few cytokines (Figure 2a, green line) did not properly belong to either class. These included IL-10, IL-1β, IL-15 and two IFN-α isoforms. Because several of these cytokines have previously been associated with the alternative pathway, we referred to this class as alternative II. Not surprisingly [5], MPs stimulated with TNF-α (Figure 2a, purple line) and, most dramatically, TNF-β and GM-CSF (Figure 2a, black line) had a totally independent effect on the transcriptional regulation of LPS-induced CD14^+ ^MPs [5].

Cytokines classified according to the previous groups were tested for class prediction by applying unsupervised principal component analysis (PCA) to the global, unfiltered 17K gene dataset (Figure 2b). This analysis independently classified cytokines in two groups corresponding to the alternative (Figure 2b, blue circles) and classical (Figure 2b, red circles) cytokine classes. Most of the cytokines belonging to the alternative II class (Figure 2b, green circles) grouped with the alternative group, whereas TNF-α (Figure 2b, purple circle and arrow), TNF-β and GM-CSF (Figure 2b, gray circles) remained separate. The sample treated with LPS alone (Figure 2b, yellow circle and arrow) grouped with the classical cytokines, confirming the predominant pro-inflammatory effects of this bacterial product and its alignment with the classical pathway of MP activation [1,11]. This finding based on the complete dataset indicates the intrinsic bias of this study aimed at exploring the alternative modulation of the MP response to LPS.

Particular mention should be made of the erratic behavior of various IFN-α subtypes, which clustered indiscriminately between the two main cytokine classes. Interestingly, however, alignment of the IFN-α protein sequences through the EMBL-EBI Clustal W database identified, with the exception of IFN-αG, a close relationship among the IFN-α subtypes that clustered with the alternative type of cytokines (data not shown). This subclassification was also supported by the phylogenetic relationship among interferons described by Henco [13]. This information suggests that specific domains of the IFN-α molecules may have dramatically different effects in the modulation of the MP response to pathogen [14,15]. Interestingly, IFN-α_2_, which is the one most commonly used in clinical trials as a pro-inflammatory cytokine, clustered with the classical cytokines adjacent to IFN-γ.

### Cytokine-mediated modulation of LPS-stimulated CD14^+ ^MPs predominantly affects pathways downstream of NFκB

**Figure 3:**
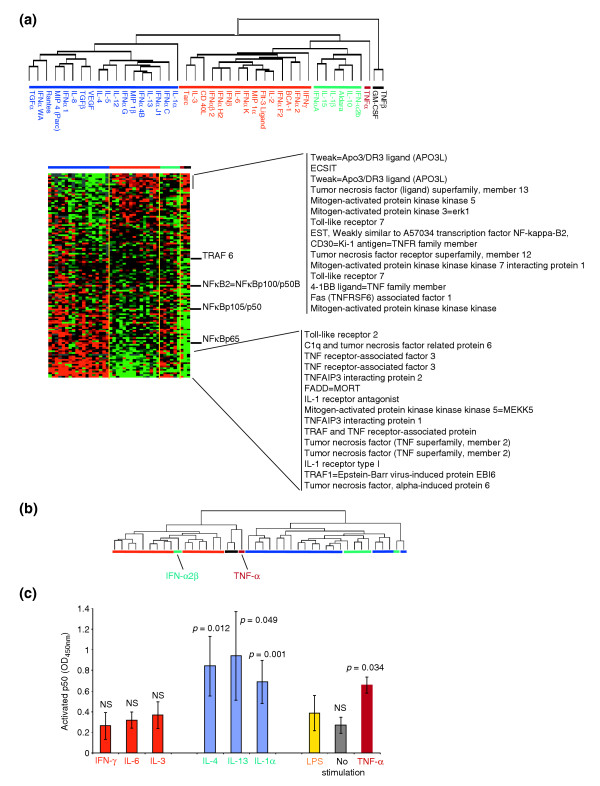
Effect of cytokines on the expression of genes dependent on NFκB activation. **(a) **One hundred and twenty-one genes associated with the downstream effects of NFκB activation were selected from the unfiltered 17K gene dataset and reclustered without changing the cytokine treatment grouping as per Figure 2. The genes most significantly different in expression between the two classes are listed on the right. **(b) **All samples were reclustered (dendrogram) using the 121 genes described in (a). Color coding is identical to that in Figure 2. **(c) **The histogram depicts detection of the free NFκB subunit p50 in four consecutive experiments. *p *= *p*_2 _value, NS, nonsignificant. Blue bars, MPs stimulated with LPS (1 h) + alternative cytokines (additional 30 min stimulation); red bars, classical cytokines; yellow bar, MPs stimulated with LPS alone; gray bar, unstimulated MP control; purple bar, TNF-α alternative II class of cytokines.

Signatures associated with several pathways of immune-cell activation were constructed by selecting genes from the global pool of 17K clones according to literature information without pre-existing information about the association of their expression to either class of cytokines. Signature genes were then subjected to supervised clustering according to the cytokine classification shown in Figure 2a. This independent process identified virtual signatures, in some cases portraying opposite transcriptional regulation by the two classes. The signature that most strongly discriminated the two classes comprised 121 genes whose expression is closely dependent on NFκB modulation [11] (Figure 3a). This is not surprising as LPS acts through engagement of Toll receptor 4 (TLR4) and CD14, with resulting activation of NFκB [3]. It would, therefore, seem intuitive that the strongest modulation in the present experimental conditions would target this pathway. In particular, several TNF- and IL-1-related genes classically modulated by NFκB during the acute phases of the innate immune response [11] were strongly and inversely modulated by the two cytokine classes.

The same 121 genes were used for unsupervised class prediction by reclustering cytokine-treated samples (Figure 3b). This independent analysis segregated cytokines into two classes that with the exception of one (IFN-α2b) matched the respective original classical and alternative classification (Figure 3a, red and blue horizontal bars, respectively). Interestingly, the cytokines that belonged to the alternative II class clustered with the alternative cytokines (Figure 3a, green horizontal bars) while TNF-α (Figure 3a, purple horizontal bar), TNF-β, and GM-CSF (Figure 3a, dark gray horizontal bar) clustered separately but in proximity of the classical group. Analysis of early signaling events occurring 1 hour and 30 minutes after LPS stimulation and, therefore, 30 minutes after the additional cytokine exposure, demonstrated significantly increased levels of the free p50 subunit of NFκB in MP whole-cell extracts treated with alternative class cytokines (IL-4 and IL-13). In addition, IL-1α and TNF-α significantly upregulated p50, whereas no significant changes were caused by classical cytokines (IFN-γ, IL-6 and IL-3). Extracts from MPs stimulated only with LPS also failed to demonstrate changes in NFκB subunit release; this is probably related to the 90-minute period from stimulation that allowed a return of signaling molecules to baseline conditions (Figure 3c). The similarity of the IL-1α and TNF-α effects on p50 to those of alternative cytokines contrasts with the dramatic differences observed on the respective transcriptional profiles, suggesting that other pathways induced by these cytokines may prevail in the conditions tested here.

Among the additional pathways tested, those mediated through STATs, Janus kinases (JAKs), and interferon regulatory factor (IRF) did not appear consequential to the experimental conditions tested in this study (data not shown).

### Cytokine-mediated modulation of metalloproteinase expression in LPS-stimulated CD14^+ ^MPs

**Figure 4:**
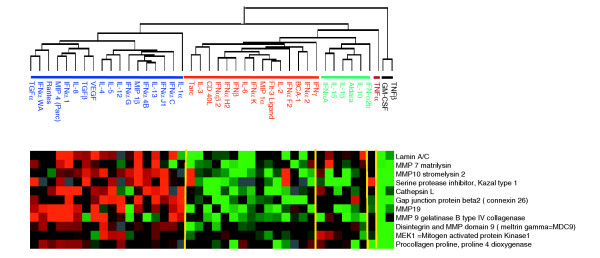
Effect of cytokines on the expression of genes for matrix metalloproteinases (MMPs) and MMP-related genes. MMP genes and MMP-related genes (such as disintegrins, IFN-γ and related genes, IFN-α, α-defensins, TGF-β, TNF-α, IGF-1, EGF, FGF, IL-1, MCP-3 - complete list available from the authors on request) were clustered according to the alternative and classical groups and filtered for 70% presence and at least one value equal to or greater than a 1.5-fold change in expression (a total of 36 genes). The clusterogram displays 11 of the most representative genes. Color coding is as Figure 2.

Matrix metalloproteinases (MMP) are tightly connected to MP activation. MMP released by MPs contribute to normal and pathological tissue remodeling and MP migration. In addition MMP function as regulatory proteins by promoting the activation or degradation of cytokines. Finally, MMP are susceptible to cytokine stimulation. Thirty-six MMP and MMP-related genes (filtered from a larger group of 184 MMPs, disintegrins, α-defensins, TGF-β, TNF-α, insulin-like growth factor 1 (IGF-1), epidermal growth factor (EGF), fibroblast growth factor (FGF), IL-1 and monocyte chemotactic protein-3 (CCL7/MCP-3) genes) [16,17] were clustered according to the alternative and classical group denomination and the 11 most representative are shown in Figure 4. The alternative group of cytokines induced the transcription of MMPs (MMP 7, 9, 10, 19), enzymes related to MMP function (disintegrin ADAM 9, pro-collagen proline dioxygenase, MEK1 kinase, serine protease inhibitor, cathepsin L) and structural proteins (gap junction connexin 26, laminin A/C). This confirms the role of alternative MP activation in promoting tissue remodeling, cell-cell interactions and local control of the inflammatory process through activation (via MMP-7, 9) [18-21] or degradation (via MMP-19) of cytokine activity [17,22]. In addition, these observations suggest a role for the cytokines in the alternative group (other than the well documented IL-4 or IL-13) in polarizing MPs toward an M2 regulatory phenotype.

### Characterization of the alternative and classical groups of cytokines

**Figure 5:**
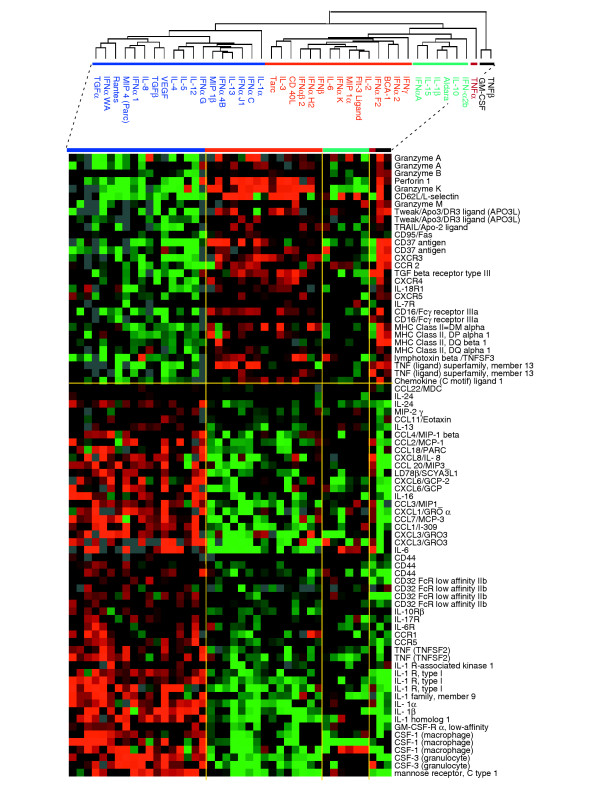
Selection of genes differentially expressed between classically and alternatively activated MPs based on previous annotations linking their function to MP activation. Eighty-one representative genes with known MP-associated function are shown among 2,007 genes differentially expressed (*p*_2 _< 0.001, Student's *t*-test). The nomenclature for chemokines and chemokine receptors follows the recommendations of the IUIS/WHO subcommittee on chemokine nomenclature [44]. Color coding as in Figure 2.

Comparison of gene-expression patterns induced by the two cytokine classes (classical and alternative) identified 2,007 genes that were differentially expressed at a less than 0.001 significance level (*t*-test, p_2_-value). Genes associated with immune function were proportionally over-represented. A selection of genes relevant to MP function is shown in Figure 5. In most cases, the pattern of expression echoed the class allocation suggested by the literature [23]. Classical cytokines induced genes responsible for the cytotoxic and migratory properties of MPs such as those for CD95, TRAIL, granzymes, perforin, CD16 (stimulatory Fcγ receptor) and CD62L. In addition, antigen presentation was enhanced as suggested by the coordinate expression of several HLA class II genes. IFN-γ and the other classical cytokines inhibited the expression of the macrophage-derived chemokine CCL22/MDC, while upregulating the expression of CXCR3, as previously reported [23].

Alternative cytokines induced the expression of several cytokines and their respective receptors involved in the chemotaxis and activation of neutrophils, MPs, natural killer (NK) cells, DCs, helper T lymphocytes (Th2) and B lymphocytes. Several genes known to be associated with alternative MP activation [5,23] were consistently upregulated. These included the mannose receptor, the inhibitory Fc-IIb receptor CD32, and the cell-surface molecule CD44 [24], which is associated with the disposal of inflammatory cell corpses without expansion of the inflammatory process. Several inducible chemokines were expressed in response to alternative stimulation such as CCL22/MDC, supporting the emerging role of this cytokine as an enhancer of polarized Th2 responses [25]. Other chemokines known to be induced by master type II cytokines and associated with the induction of Th2 responses were also induced by alternative activation; these included CCL11 (eotaxin), CCL1 (I-309), CCL2 (MCP-1) and CCL7 (MCP-3) [23]. Of interest was the relatively higher expression of IL-24, a cytokine belonging to the IL-10 family constitutively expressed by MPs [26]. Although the true role of this cytokine in inflammatory processes is not known, it is likely that its pro-apoptotic and angioregulatory properties have important roles in tissue repair and remodeling during inflammation. Finally, various chemokine receptors responsible for MP trafficking and localization were differentially regulated by the two cytokine groups, including in particular CCR1 and CCR5, which were induced by alternative stimulation, and CCR2, induced by classical stimulation.

This early transcriptional profile underlines the primary role of MPs during the acute phases of the response to pathogen as effector cells that can kill pathogens, take up antigen and migrate to local regional lymph nodes to recruit adaptive immune responses (classical activation). In contrast, alternative cytokines may be produced in the microenvironment to maintain a resident MP phenotype rich in chemokine production, which can attract Th2-type immune responses while continuing pathogen clearance through retention of phagocyte properties and promoting tissue remodeling.

Surprisingly, genes of the IL-1 family and its receptors, TNF and IL-6 were consistently upregulated by the alternative class of cytokines, suggesting that LPS alters MP polarization with regard to these cytokines [5,10]. Particularly interesting is the alternative induction of IL-6 and its receptor, which may play a central role in mediating the transition from neutrophils to MP recruitment during progression from acute to chronic inflammation [27].

### Validation of microarray analysis by TaqMan real-time PCR

**Figure 6:**
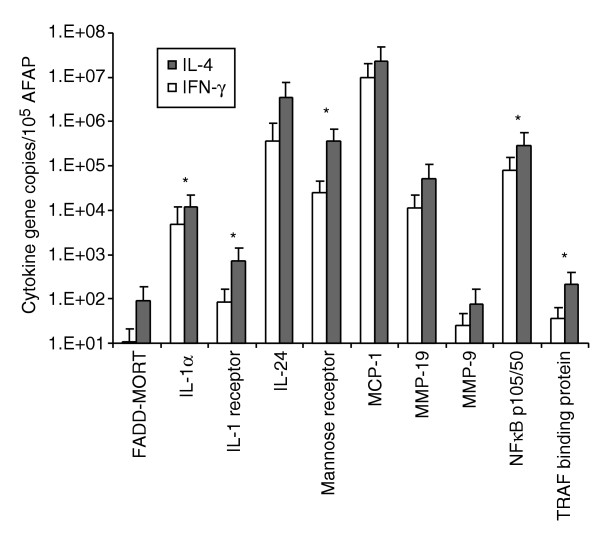
Induction of gene expression by stimulation of LPS-activated MPs with IFN-γ and IL-4. MPs obtained from PBMC from five normal donors were stimulated for 4 h with one cytokine representative of the alternative (IL-4) and one of the classical group (IFN-γ) and gene transcription measured by TaqMan real-time PCR. The relative quantification of 10 genes was calculated by normalizing the ratio of the mean copy number for each gene with the mean copy number of the reference AFAP gene in MPs from five donors. Statistically significant differences (*p*-value < 0.05) between the two cytokine treatments as assessed by Student's *t*-test are represented by an asterisk.

To define the validity and accuracy of our global microarray analysis, quantitative TaqMan real-time PCR was performed on amplified RNA material isolated after stimulation of monocytes obtained from five additional normal donors with representative cytokines/soluble factors selected from the alternative and from the classical groups. Comparison of monocyte stimulation with a candidate cytokine from the alternative/M2 (IL-4) and the classical/M1 (IFN-γ) group, respectively, is shown in Figure 6. Ten genes whose expression was upregulated by alternative cytokines were tested, as the thrust of the analysis was the evaluation of the effect of alternative cytokines on LPS-stimulated MPs. The relative expression of five genes out of 10 (IL-1α, IL-1 receptor, mannose receptor, NFKb-p105/50 and TRAF) was significantly higher after treatment with IL-4, as suggested by the array data. Also, the expression profiles of the other genes tested reproduced the pattern observed in the array experiments even if they did not reach statistical significance for each individual gene. This is not surprising because array experiments summarize in signature fine differences in gene expression, that often describe patterns rather than absolute significance for individual genes.

**Figure 7:**
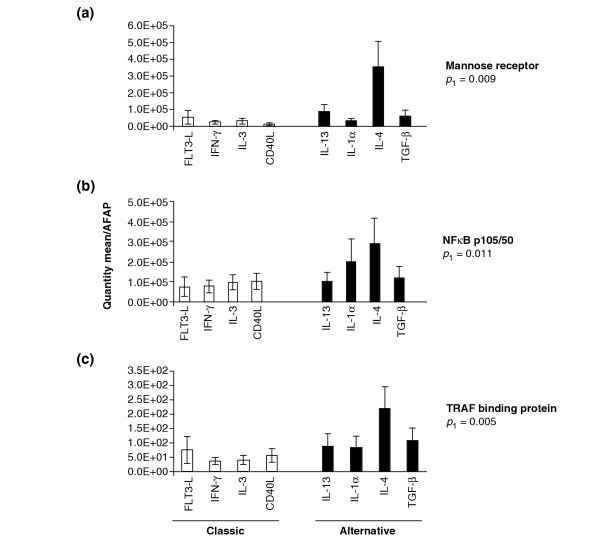
Induction of gene expression by cytokines of the alternative and classical groups. MPs derived from PBMC from five normal donors were stimulated for 4 h with four cytokines/soluble factors selected from the alternative group (IL-13, IL-1α, IL-4, TGF-β) and four from the classical group (FLT-3 L, IFN-γ, IL-3, CD40L). Gene expression was assessed by TaqMan real-time PCR. Relative estimates of **(a) **mannose receptor, **(b) **NFκB and **(c) **TRAF binding protein genes were calculated by averaging the ratio of the quantity mean of each gene normalized to the reference AFAP gene in five donors. Statistical significance is expressed as Student's *t*-test *p*-value.

Reproducibility of the estimates of mannose receptor, NFKb-p105/50 and TRAF gene expression in MPs obtained from the same five donors was evaluated after stimulation with four cytokines/soluble factors selected from the alternative (IL-13, IL-1α, IL-4, TGF-β) and four from the classical groups (FLT-3 ligand, IFN-γ, IL-3, CD40L) (Figure 7). In all cases gene expression significantly reflected the pattern of gene expression detected by microarray analysis (Figures 3, 5).

## Discussion

It has been suggested that MP activation and maturation progresses through a polarized mechanism whereby two extreme products result that promote inflammation on one side and tissue repair on the other [1,5]. The first mechanism has been called the classical pathway of MP activation. It induces M1 monocytes specialized for pathogen killing and activation of innate and adaptive immune effector cells. A second, alternative, pathway induces M2-type monocytes committed to clearing pathogen through internal metabolism while reducing inflammation. This process limits the collateral damage induced by an excessive immune response, and, upon cessation of the pathogenic stimulus, promotes tissue repair. This dichotomy is based on the study of a few cytokines deemed representative of the classical mode (LPS, IFN-γ) or the alternative mode (IL-4, IL-13) of MP activation. Other cytokines such as IL-10, TGF, M-CSF, IFN-α/β and TNF, although partially overlapping both pathways, display functional effects that diverge significantly enough that they would be inaccurately grouped in either class [5].

In this study we evaluated whether MP commitment is dependent on an early bipolar switch through which most cytokines operate. This was done by testing in parallel a library of 42 stimulatory molecules possibly present in the tissue microenvironment following a pathogenic insult. This modulation was tested on MPs triggered by a pathogenic stimulus, exemplified in this case by LPS. This was done on the assumption that in most circumstances resident or migratory MPs reaching an infected area are exposed concomitantly to a pathogen and to the cytokine milieu resulting from the infection. The transcriptional profile identified by this study cannot distinguish between the direct effect of each soluble factor analyzed and the downstream activation of transcriptional pathways by secondary paracrine or autocrine secretion of biological modifiers by MPs. However, the main goal of this study was to identify the overall effect on MPs of the exposure to individual cytokines. Future analyses, focused on specific cytokine patterns described here, should possibly include the addition of blocking antibodies to segregate secondary from primary MP responses.

The results suggest that MP activation is in most cases a bipolar process regulated by an internal switch through which cytokines modulate the *yin *and *yang *of the MP transcriptional program. In fact, most cytokines preferentially induced one or other pattern of transcriptional activation. We, therefore, mapped most of the cytokines within a classical or an alternative classification according to their effects on CD14^+^, LPS-induced MPs. In particular, it appeared that transcriptional programs down-stream of NFκB activation [11] were mostly associated with either class, suggesting that the NFκB system is at the center of the switch regulating MP activation/differentiation in the conditions tested in the present study. This is not surprising as LPS signaling is mediated through the TLR-4 and CD14, which in turn directly regulate the IκB kinase (IKK)-NFκB pathway [3,11,28,29]. It appears that cytokine regulation modulates the release of the p50 subunit of NFκB, whch is in turn responsible for the downstream effects on the transcriptional program. Interestingly, reclustering of cytokines based on NFκB-dependent genes consolidated the two classes of cytokines adding TNF-α and TNF-β, IFN-α_2b _and GM-CSF to the classical group and the alternative type II cytokines with the alternative group, suggesting that NFκB may be central to MP polarization.

This information cannot of course be generalized to other conditions as the model tested is strongly biased by NFκB induction by LPS. This is underlined by the unexpected alternative upregulation of genes associated with IL-1, TNF and IL-6 [11]. This observation suggests that in different conditions, cytokines may differently modulate MP activation, possibly through various modulatory feedback mechanisms [5]. In addition, genes associated with the interrelated arginine and tryptophan pathways that modulate nitric oxide induction and are indirectly associated with NFκB function were, at least in part, differentially regulated by the two classes of cytokines [30-32]. It has been suggested that inducible nitric oxide is produced rapidly after LPS stimulation of MPs and inhibits NFκB through the stabilization of IκB [16]. It is possible that cytokines may counteract this effect through modulation of this metabolic junction.

Cytokine and chemokine effects on MP function are tightly intertwined with the enzymatic activities of MMP. Membrane-bound cytokine receptors and adhesion molecules can be released from the cell surface by MMPs acting as 'sheddases' or 'convertases'. This, in turn, can downregulate cell-surface signaling by removal of receptors, or induce paracrine activity by release of soluble proteins. Not surprisingly, IL-13 overexpression results in production of several MMPs [33]. For instance, MMP-9 activates latent TNF-α on the surface of MPs or soluble VEGF [19,34]. Furthermore, Yu and Stamenkovic [21] observed that gelatinase B/MMP9 bound to CD44 activates latent TGF-β stored in the pericellular matrix. MMP-7 can enhance tissue repair by facilitating migration of epithelial cells [16]. In agreement with these reports we observed that the transcription of MMP-7, MMP-9 and CD44 are coordinately induced (Figures 4, 6) by the alternative activation of MPs. Conversely, the downregulation of MMP-7 and MMP-9 by the classical cytokines confirms their inhibitory effects on MMP expression. Inhibition of MMP-9 production by IFN-β and IFN-γ has been recently reported by Sanceau *et al*. [35], who noted that interferons regulate MMP expression through IRF/NFκB interaction. Binding of NFκB p50/p65 to the MMP-9 promoter is competitively inhibited by IFN-β and IFN-γ-induced IRF-1. Possibly, NFκB regulation of MMP promoters through release of p50 (Figure 3) was responsible for the transcriptional activation of MMP expression by alternative cytokines observed in this study (Figure 4). These observations confirm the tight specificity of the relationship between cytokine and MMP regulation, which is finely toned at several check points and strongly polarized, in these experimental conditions, toward an M2 phenotype.

Enhanced transcription of gap junction/connexin 26 by the alternative cytokines is also of particular interest in view of the hypothesized junctional communications among MPs or between MPs and endothelial cells [36,37]. The finding that MPs activated by alternative cytokines induce the transcription of genes for gap junction components is of physiological importance and may be the missing link in the identification of factors that regulate the expression of gap junction connexins in MPs. In addition, it opens up the possibility that in a milieu dominated by alternative cytokines, where the ultimate goal is to return to homeostasis, the induction of gap junctions increases the ability to transmit or receive regulatory signals [38] that could facilitate the return to normal housekeeping functions.

## Conclusions

The early-phase transcriptional profile presented in this study may not comprehensively parallel the plethora of biological effects that a given cytokine can induce under *in vitro *or, most importantly, *in vivo *conditions. Secondary, autocrine and paracrine modulation through the cytokine network following a primary stimulation may introduce novel on and off switches that could override the original signal. Nevertheless, this analysis is directly informative on the primary effect of individual cytokines on the early stages of LPS stimulation and, therefore, may be most informative on the way MP maturation may be polarized at the early stages of the immune response. The clustering of most cytokines into two main groups suggests that their control of central switches (NFκB), or regulatory molecules (cytokines, MMPs, gap junctions, cytotoxic molecules, migratory markers) is essentially bimodal. This polarization program turns MPs to a 'cytotoxic' or a 'symbiotic' phenotype [18]. In physiologic conditions, this dualism is probably modulated by a multiplicity of factors: the extent and duration of the environmental insult and the conditions of the resulting microenvironment. Possibly, predominant and persistent stimulation by pathogen components (such as LPS) may polarize MP towards the cytotoxic phenotype. A predominantly regulatory response is then mounted by the host, mediated by alternative cytokines that would take over at a later stage to induce a symbiotic phenotype aimed at resuming homeostasis upon pathogen clearance.

## Materials and methods

### MP separation and FACS staining

Peripheral blood mononuclear cells (PBMCs) from an HLA-A*0201-positive healthy caucasian male donor age 35 were collected at the Department of Transfusion Medicine, NIH. PBMCs were isolated by Ficoll gradient separation and frozen until analysis. After thawing, PBMCs were kept overnight in 175-cm^2 ^tissue-culture flasks (Costar) in complete medium (CM) consisting of Iscove's medium (Biofluids) supplemented with 10% heat-inactivated human AB-serum (Gemini Bioproducts), 10 mM HEPES buffer (Cellgro; Mediatech), 0.03% l-glutamine (Biofluids), 100 U/ml penicillin/streptomycin (Biofluids), 10 μg/ml ciprofloxacin (Bayer), and 0.5 mg/ml amphotericin B (Biofluids). Adherent and non-adherent cells were gently removed from the flask and centrifugated. MPs were separated by negative selection using the MP isolation kit and an autoMACS system (Miltenyi). Before and after separation cells were stained with anti-CD14-FITC (Becton Dickinson), and analyzed using a FACScalibur flow cytometer and CellQuest software (Becton Dickinson).

### Stimulation of MPs and RNA isolation

Negatively selected CD14^+ ^cells were washed twice with serum-free OPTI-MEM (OM) medium (Gibco-BRL) prepared similarly to CM. CD14^+ ^cells were then seeded at a concentration of 1 × 10^6^/ml in 10 ml OM in 25 cm^2 ^flasks (Falcon) and stimulated with 5 μg/ml LPS (Sigma) for 1 h. LPS was used at 5 μg/ml to simulate maximal pathogen exposure as in [6]. No LPS was added to the non-stimulation control flask. After 1 h, 42 cytokines, chemokines and soluble factors were added individually to the MP suspensions (Table 1). Then, 4 and 9 h after LPS stimulation, MPs were harvested, washed twice in PBS and lysed for RNA isolation using 700 μl RNeasy lysis buffer (Qiagen) per 25 cm^2 ^flask, according to the manufacturer's protocol.

### Probe preparation, amplification and hybridization to microarrays

Total RNA was isolated using RNeasy minikits (Qiagen). Amplified antisense RNA (aRNA) was prepared from total RNA (0.5-3 μg) according the protocol previously described by us [9,39]. Test samples were labeled with Cy5-dUTP (Amersham) while the reference sample (pooled normal donor PBMCs) was labeled with Cy3-dUTP. Test-reference sample pairs were mixed and co-hybridized to 17K cDNA microarrays.

### Microarrays and statistical analyses

Hybridized arrays were scanned at 10-μm resolution on a GenePix 4000 scanner (Axon Instruments) at variable PMT voltage to obtain maximal signal intensities with less than 1% probe saturation. Resulting jpeg and data files were analyzed via mAdb Gateway Analysis tool [40]. Data were further analyzed using Cluster and TreeView software [12] and Partek Pro software (Partek). The global gene-expression profiling of 4- and 9-h treated and untreated MP consisted of 98 experimental samples. Subsequent low-stringency filtering (80% gene presence across all experiments and removal of genes that did not have a log_2 _≥ 1.2: 2.3 ratio in at least one of the samples) selected 10,370 genes for further analysis. Clustering of experimental samples according to Eisen *et al*. [12] was based on these genes. Gene ratios were average corrected across experimental samples and displayed according to the central method for display using a normalization factor as recommended by Ross [41].

### NFκB protein activation analysis

MPs separated from peripheral blood by adherence were stimulated for 1 h with LPS and for an additional 30 min with cytokines selected from the alternative group (IL-4, IL-13, IL-1α) or the classical group (IFN-γ, IL-6, IL-3). In addition, TNF-α was tested. After 90 min stimulation, cytoplasmic cell extracts were isolated using a cytoplasmic and nuclear extract kit (Active Motif), and the TransAM NFκB transcription factor kit (Active Motif) was used to detect activation of NFκB subunits p50, p52, p65, c-Rel and RelB, according to the manufacturer's protocol.

### Real-time quantitative RT-PCR

MPs obtained from PBMC of five normal caucasian donors (three males, two females, age range: 35-55 years old) were stimulated with four cytokines/soluble factors selected from the alternative group (IL-13, IL-1α, IL-4, TGF-β) and four from the classical groups (FLT-3L, IFN-γ, IL-3, CD40L). TaqMan real-time PCR was performed on amplified RNA material isolated after stimulation for 4 h in conditions identical to those applied for the cDNA array study to validate the expression of the following 10 genes: TRAF binding protein, NFκB-p105/50, MMP9, MMP19, MCP-1, mannose receptor, IL-24, IL-1R, IL-1A and FADD-MORT. An ABI Prism 7900 HT sequence detection system with 384-well capability (Applied Biosystems) was used for detection. Primers and TaqMan probes (Biosource) were designed to span exon-intron junctions and to generate amplicons of less than 150 bp. TaqMan probes were labeled at the 5' end with the reporter dye molecule FAM (6-carboxyfluorescein; emission λ_max _= 518 nm) and at the 3' end with the quencher dye molecule TAMRA (6-carboxytetramethylrhodamine; emission λ_max _= 582 nm). The following are the sequences for forward (f) and reverse (r) primer and probe (p) pairs:

IL-1α f: TGTATGTGACTGCCCAAGATGAA

IL-1α r: ACTACCTGTGATGGTTTTGGGTATC

IL-1α p: FAM-AGTGCTGCTGAAGGAGATGCCTG-TAMRA

IL-1 rec. f: TGTCACCGGCCAGTTGAGT

IL-1 rec. r: GCACTGGGTCATCTTCATCAATT

IL1 rec p: FAM-ACATTGCTTACTGGAAGTGGAATGGGTCAG-TAMRA

TRAF bp f: TTGCTTACAG AGGTGTCTCAACAAG

TRAF bp r: CTCCGGATTTGTTCTGTCAGTTC

TRAF bp p: FAM-AGCAAAGTGTATTCCAGCAATGGTGTGTCC-TAMRA

MMP9 f: TGGATCCAAAACTACTCGGAAGA

MMP9 r: GAAGGCGCGGGCAAA

MMP9 p: FAM-CGCGGGCGGTGATTGACGAC-TAMRA

MMP19 f: GACGAGCTAGCCCGAACTGA

MMP19 r: TTTGGCACTCCCGTAAACAAA

MMP19 p: FAM-TCAGCAGCTACCCCAAACCAATCAAGG-TAMRA

Mannose receptor f: CTAAACCTACTCATGAATTACTTACAACAAAAG

Mannose receptor. r: CTCCGGCCACGTTGGA

Mannose receptor p: FAM-ACACAAGGAAGATGGACCCTTCTAAACCGTC-TAMRA

FADD-MORT f: GGTGGCTGACCTGGTACAAGA

FADD-MORT r: ACATGGCCCCACTCCTGTT

FADD-MORT p: FAM-TTCAGCAGGCCCGTGACCTCCA-TAMRA

NFκB p105/50 f: CTACACCGAAGCAATTGAAGTGA

NFκB p105/50 r: CAGCGAGTGGGCCTGAGA

NFκB p105/50 p: FAM-CAGGCAGCCTCCAGCCCAGTGA-TAMRA

IL-24 f: AAGAAAATGAGATGTTTTCCATCAGA

IL-24 r: CTGTTTGAATGCTCTCCGGAAT

IL-24 p: FAM-ACAGTGCACACAGGCGGTTTCTGC-TAMRA

MCP-1 f: CATGGTACTAGTGTTTTTTAGATACAGAGACTT

MCP-1 r: TAATGATTCTTGCAAAGACCCTCAA

MCP-1 p: FAM-AACCACAGTTCTACCCCTGGGATG-TAMRA

Standards for the selected genes were amplified by reverse transcriptase primer-specific amplification of 6 μg antisense RNA obtained from PBMCs stimulated *in vitro *with IL-2 (300 IU/ml and Flu M1 peptide) and reverse transcribed using random dN_6 _primers (Boehringer Mannheim). Amplified cDNA standards were quantified by spectrometry and the number of copies was calculated using the Oligo Calculator software [42]. Six micrograms of test antisense RNA samples were converted to cDNA using random primers and were immediately used for quantitative real-time PCR (RT-PCR). RT-PCR reactions of cDNA samples were conducted in a total volume of 20 μl, including 1 μl cDNA, 1x TaqMan Master Mix (Applied Biosystems), 2 μl of 20 μM primers and 1 μl of 12.5 μM probe. Thermal cycler parameters included 2 min at 50°C, 10 min 95°C and 40 cycles involving denaturation at 95°C for 15 sec, annealing-extension at 60°C for 1 min.

Linear regression analyses of all standard curves were 0.98 or greater. Standard curve extrapolation of copy number and quantity means were performed using the ABI Prism SDS 2.1 software (Applied Biosystems). Normalization of samples was performed by dividing the quantity mean of the gene of interest run in duplicate by the quantity mean of reference actin filament associated protein (AFAP) gene × 10^5 ^[43].

## Additional data files

The following additional data are available with the online version of this article. Additional data file 1 is a spread sheet containing microarray raw data (intensity ratios of test versus reference samples) subsequently subjected to cluster analysis.

## Supplementary Material

Additional data file 1 A spread sheet containing microarray raw data (intensity ratios of test versus reference samples) subsequently subjected to cluster analysis Click here for additional data file

## Figures and Tables

**Table 1:** Concentration and doses of cytokines/soluble factors used for stimulation of monocytes

Cytokine	Concentration *in vitro*	Source
Aldara	3 μM	3M Pharmaceuticals
BCA-1	10 ng/ml	Peprotech
CD40 L	500 ng/ml	Peprotech
FLT-3 ligand	100 ng/ml	Peprotech
GM-CSF	1,000 IU/ml	Peprotech
IFN-γ	1,000 U/ml	Biogen
IFN-α2	1,000 U/ml	Peprotech
IFN-α2b (α2, α2b,)	1,000 U/ml	PBL Biomedical Laboratories
IFN-αA (2a)	1,000 U/ml	PBL Biomedical Laboratories
IFN-αI	1,000 U/ml	PBL Biomedical Laboratories
IFN-αB2	1,000 U/ml	PBL Biomedical Laboratorios
IFN-α4b	1,000 U/ml	PBL Biomedical Laboratories
IFN-αC	1,000 U/ml	PBL Biomedical Laboratories
IFN-αF	1,000 U/ml	PBL Biomedical Laboratories
IFN-αG	1,000 U/ml	PBL Biomedical Laboratories
IFN-αH2	1,000 U/ml	PBL Biomedical Laboratories
IFN-αJ1	1,000 U/ml	PBL Biomedical Laboratories
IFN-αK	1,000 U/ml	PBL Biomedical Laboratories
IFN-αWA	1,000 U/ml	PBL Biomedical Laboratories
IFN-β	1,000 U/ml	PBL Biomedical Laboratories
IL-1α	10 ng/ml	National Cancer Institute (NCI) Biological Research Branch
IL-1β	10 ng/ml	NCI Biological Research Branch
IL-2	6,000 IU/ml	Chiron
IL-3	15 ng/ml	NCI Biological Research Branch
IL-4	1,000 IU/ml	Peprotech
IL-5	10 ng/ml	Peprotech
IL-6	100 ng/ml	Peprotech
IL-8	100 ng/ml	Peprotech
IL-10	10 ng/ml	Peprotech
IL-12	10 ng/ml	Peprotech
IL-13	20 ng/ml	Peprotech
IL-15	20 ng/ml	Peprotech
LPS only	5 μg/ml	Sigma Aldrich
MIP-1α (CCL3)	100 ng/ml	Peprotech
MIP-4 (PARC, CCl18)	100 ng/ml	Peprotech
MIP-1β (CCL4)	100 ng/ml	Peprotech
RANTES (CCL5)	100 ng/ml	Peprotech
TARC (CCL17)	100 ng/ml	Peprotech
TGF-α	10 ng/ml	Peprotech
TNF-β	20 ng/ml	Peprotech
TGF-β	5 ng/ml	Peprotech
TNF-α	100 ng/ml	Peprotech
VEGF	10 ng/ml	R&D Systems
